# A Transfer Learning Approach for Toe Walking Recognition Using Surface Electromyography on Leg Muscles

**DOI:** 10.3390/s25051305

**Published:** 2025-02-20

**Authors:** Andrea Manni, Gabriele Rescio, Anna Maria Carluccio, Andrea Caroppo, Alessandro Leone

**Affiliations:** National Research Council of Italy, Institute for Microelectronics and Microsystems, 73100 Lecce, Italy; annamaria.carluccio@imm.cnr.it (A.M.C.); andrea.caroppo@cnr.it (A.C.); alessandro.leone@cnr.it (A.L.)

**Keywords:** gait, toe walking, transfer learning, sEMG, sensors

## Abstract

Gait is a complex motor process that involves the coordination and synchronization of various body parts through continuous interaction with the environment. Monitoring gait is crucial for the early detection of abnormalities, such as toe walking, which is characterized by limited or absent heel contact with the floor during walking. Persistent toe walking can cause severe foot, ankle, and musculature conditions; poor balance; increased risk of falling or tripping; and can affect overall quality of life, making it difficult, for example, to participate in sports or social activities. This study proposes a new approach to detect toe walking using surface Electromyography (sEMG) on lower limbs. sEMG sensors, by measuring the electrical activity of muscles, can see signals before the movement corresponding to muscle activation, contributing to an early detection of a possible problem. The sEMG signal presents significant complexity due to its noisy nature and the challenge of extracting meaningful features for classification. To address this issue and enhance the model’s robustness across different devices and configurations, a Transfer Learning (TL) approach is introduced. This method leverages pre-trained models to effectively handle the variability of sEMG data and improve classification accuracy. In particular, Continuous Wavelet Transform (CWT) is applied to sEMG-filtered signals (with time windows of 1 s) to convert them into 2D images (scalograms). Preliminary tests were performed on a public dataset using some of the most well-known pre-trained architectures, obtaining an accuracy of about 95% on InceptionResNetV2.

## 1. Introduction

Gait assessment is a critical research field in biomechanics and clinical diagnostics, involving the analysis of walking patterns to diagnose, treat, and monitor various conditions. Gait abnormalities, such as toe walking, characterized by the absence or limitation of heel contact with the ground during the walking cycle, can have a serious impact on physical and social well-being. Persistent toe walking is associated with musculoskeletal complications, increased risk of falls, and limitations in daily and recreational activities [1]. This paper aims to focus on the detection of toe walking commonly observed in both children and adults. It can arise from various causes, including idiopathic factors or underlying neurological and orthopedic conditions, such as Achilles tendon shortening or stiffness in the calf muscles. The development of hardware–software systems for early detection of these abnormalities is critical for effective intervention [2,3]. Furthermore, in addition to clinical diagnostics, such systems can find application in rehabilitation through the production of real-time biofeedback to patients to improve therapy outcomes. Meanwhile, other areas of applications may be in the industrial field through integration into wearable devices for workplace ergonomics or the sports domain for monitoring athletic performance or inappropriate postures.

Traditional observational methods are widely used in clinical settings for their simplicity [4]; however, they suffer from considerable subjectivity and depend heavily on the observer’s experience, whereas motion analysis systems, such as those that exploit optical motion capture technology, provide accurate kinematic data but require ad hoc structured environments and are not portable. These limitations make them inaccessible for routine diagnostics or for use in remote or resource-limited scenarios.

Motion capture systems are widely used for gait analysis, using high-speed, time-of-flight cameras and reflective markers to track movement in three dimensions. These systems provide accurate kinematic data, allowing clinicians to quantify joint angles and stride length and cadence, as well as identify step cycle deviations characteristic of toe walking. However, they require expensive equipment and controlled laboratory environments, which can limit accessibility and usability [5]. Another technique used relies on the use of force platforms that measure ground reaction forces during walking, providing insight into the abnormal force patterns associated with forefoot loading, balance, and stability. These platforms are very accurate and valuable for kinetic analysis, but they have the limitation of being stationary and expensive, which may limit their routine use [6].

Wearable sensors, such as accelerometers, gyroscopes, and Inertial Measurement Units (IMUs), have emerged as a portable alternative [7,8]. These devices measure spatiotemporal parameters such as stride length and walking speed while detecting plantar pressure distribution to identify the first contact with the toe [9]. Although they have reduced accuracy and can be more affected by noise than vision and environmental laboratory systems, wearable sensors have the advantage of lower cost and allow continuous monitoring in both indoor and outdoor environments and can enable early detection of an abnormal condition in walking [10,11]. Table 1 shows recent works on gait assessment with their accuracy values. As can be seen, they achieve accuracies above 90%, indicating that they are reliable for the identification of toe walking conditions.

Surface Electromyography (sEMG) is a non-invasive technology that measures the electrical activity generated by muscles during movement. Its application in gait analysis has made significant progress in recent years. For instance, a 2023 study proposed a gait cycle-inspired learning strategy for the continuous prediction of knee joint trajectory from sEMG, highlighting the effectiveness of sEMG in predicting motor intentions prior to actual movement execution [18]. In the rehabilitation field, the integration of sEMG with motion capture systems has improved the understanding of motor coordination and compensation mechanisms. A 2024 study examined the use of motion analysis technologies in telerehabilitation, emphasizing the importance of sEMG in patient assessment and monitoring [19].

These advancements highlight the growing importance of sEMG in gait analysis, neural strategies, and biomechanical applications, providing more precise and reliable tools for motor assessment and rehabilitation.

Compared to video motion analysis systems, which are highly accurate but expensive, non-portable, and require significant effort for installation, sEMG offers a more practical and cost-effective alternative. Unlike observational techniques that rely on visible movement, sEMG detects muscle activation before it becomes externally observable, enabling earlier identification of gait abnormalities such as toe walking. Additionally, when compared to wearable IMU systems, sEMG provides higher accuracy, faster model learning, and valuable insights into muscle activation patterns, offering critical diagnostic information to identify the underlying causes of the problem. However, sEMG signals are intrinsically complex, characterized by high variability, noise, and dependence on sensor placement. To address these challenges, this study integrates sEMG with Continuous Wavelet Transform (CWT), a method that converts raw temporal signals into two-dimensional scalograms. As reported in [20,21], CWT is becoming an important tool for biomedical signal analysis. In particular, it provides significant advantages in the study of EMG signals, allowing researchers to investigate various aspects of neuromuscular function in both physiological and pathological processes within the time–frequency domain. In addition, scalograms facilitate the application of image-based Deep-Learning (DL) models, which excel at complex pattern recognition. Thus, the proposed approach combines the portability of sEMG with the accuracy of Transfer Learning (TL), offering a scalable and practical solution for gait analysis. Additionally, the TL-based software framework reduces the reliance on extensive training datasets, a common bottleneck in machine-learning applications. By leveraging TL, pre-trained models such as InceptionResNetV2 are fine-tuned for the specific task of gait detection, minimizing computational overhead and accelerating model deployment. This adaptability positions the proposed method as a viable solution for real-world applications, ranging from clinical diagnostics to wearable technologies. To the best of our knowledge, there is no relevant work on toe walking detection using sEMG wearable systems suitable for long-term monitoring. The proposed approach allows both an early identification of the problem and the acquisition of additional neuromuscular information useful for a more comprehensive investigation of the disorder in both diagnosis and rehabilitation.

The remainder of this paper is organized as follows. Section 2 reports details on the used dataset and implemented algorithmic pipeline. The results are included in Section 3, while the conclusions are reported in Section 4.

## 2. Materials and Methods

**Figure 1:**
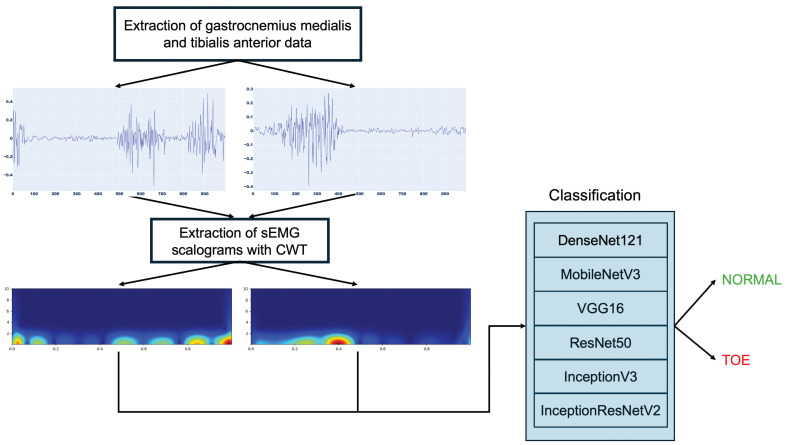
Proposed toe walking detection methodology.

In this paper, an intelligent sEMG scalogram system for toe walking detection is proposed. In the presented approach, data from two lower limb muscles involved in the execution of the activities are extracted from publicly available EMG recordings from the dataset presented in [22]. In other papers, good results were achieved using the Gastrocnemius Medialis and Tibialis Anterior muscles [23,24,25] to evaluate muscular behavior during walking. So, these muscles are also analyzed in this work, and the data extracted from these muscles are segmented into fragments of 1000 samples that are transformed into images using CWT. The resulting scalograms are sent to deep neural networks for accurate classification into normal and toe walking. Figure 1 depicts the schematic diagram of the proposed methodology.

### 2.1. Dataset

To validate the presented approach, a public dataset of kinematic, kinetic, and sEMG data [22] of human locomotion during walking, toe and heel walking, and stair ascending and descending was used. The study included 50 healthy participants (25 male, 25 female) aged 6 to 72 years (18.2–110 kg, 116.6–187.5 cm). Data were collected at the Movement Analysis Laboratory, Department of Biomedical Technology, Don Carlo Gnocchi Foundation, Milan, Italy. EMG activity was recorded unilaterally on the dominant side using an 8-channel wireless system (ZeroWirePlus, Cometa, Bareggio, Italy [26]) with self-adhesive Ag-AgCl pre-amplified electrodes (Medtronic Kendall, 24 mm diameter, 10 mm active area, 20 mm interelectrode distance). The signals were bandpass filtered (10–400 Hz) to minimize aliasing. Recordings were obtained from eight muscles: Tibialis Anterior, Soleus, Gastrocnemius Medialis, Peroneus Longus, Rectus Femoris, Vastus Medialis, Biceps Femoris, and Gluteus Maximus. For this paper, the focus was only on the Gastrocnemius and Tibial muscles to reduce the invasiveness of the system, making it suitable for long-term monitoring. Those muscles with a sampling frequency of 1000 Hz were chosen for their effectiveness in gait analysis and evaluation of lower limb muscle behavior [25,27]. The electrodes were placed according to SENIAM recommendations, and the skin was shaved, cleaned with an alcohol solution, and allowed to dry to ensure optimal signal quality. For each participant, the data acquisition session included a static calibration phase followed by a dynamic phase, which included five locomotor activities performed barefoot: walking at different speeds, toe walking, heel walking, step ascent, and step descent. The dataset is available in “.mat” format.

**Figure 2:**
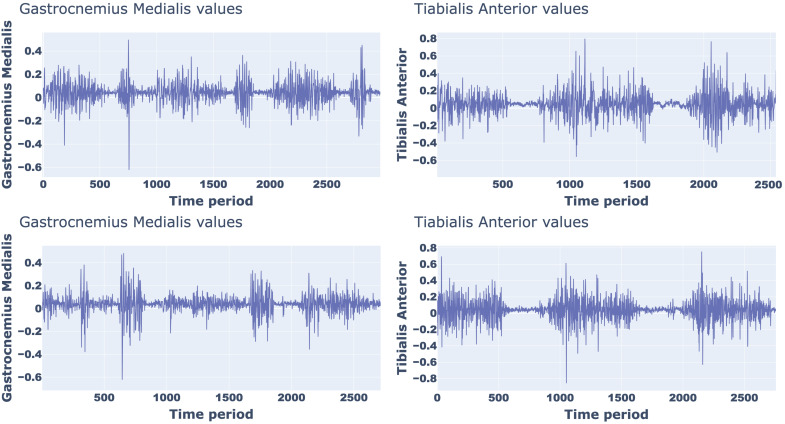
sEMG signal trend of a normal walking for two different partecipants.

As an example, Figure 2 shows the sEMG signal trend, from which the amplitude and timing of a normal walking can be appreciated, of the two muscles considered for two participants with different anthropometric characteristics (Male, 68 years, body height 179.57 cm, body mass 74.18 kg; Female, 34 years, body height 155 cm, body mass 51.63 kg).

**Figure 3:**
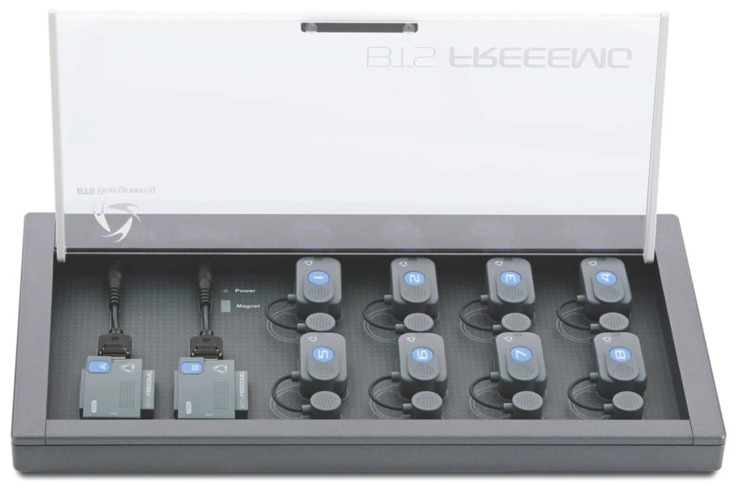
BTS Bioengineering FREEEMG1000 platform.

To evaluate the generalization capabilities of the proposed approach, an additional data collection was conducted. Specifically, five participants (three women and two men, with a mean age of 40.52 ± 6.3 years) each performed ten normal walking tests and ten toe walking tests using a commercial platform different from the one employed in the previously described dataset. Specifically, the FREEEMG1000 system (Figure 3), manufactured by BTS Bioengineering [28], was used, which is based on wireless technology and can operate using a maximum of ten lightweight and minimally invasive EMG probes. The probes are attached to pre-gelled Ag/AgCl electrodes, ensuring stable attachment, maximizing the level of usability for the user. The probes allow a direct communication with a USB receiver plugged into a processing unit. BTS probes were worn following the same procedures as described in the Public Dataset. An added advantage of the proposed hardware is the wireless design, enabling the end-user a wide spectrum of movements during task execution without limitations.

### 2.2. Pre-Processing

This phase consists of two main steps: (1) noise reduction and (2) EMG envelope extraction. In the first step, baseline noise and signal artifacts caused by sEMG electrode movement [29] are reduced. Specifically, the raw signals are filtered using a forth-order Butterworth bandpass filter with a frequency range of [20, 450] Hz. In the second step, to ensure comparability of the signals for further processing, the linear envelope of the signal is derived through full rectification followed by low-pass Butterworth filtering with a 10 Hz cutoff frequency.

### 2.3. Continuous Wavelet Transform

It is assumed that the application of TL to toe walking recognition can use data from other users to acquire general features with a pre-trained Convolutional Neural Network (CNN) prior to fine-tuning it for a new user. The application of TL can thus improve the classification performance of a new user, reducing at the same time the training effort for the user since less data are necessary to fine-tune compared to training.

Such pre-trained networks require not 1D signals but 2D images as input. To achieve this, a Time–Frequency Representation (TFR) of the sEMG signal was considered, containing more in-depth information [30]. In particular, the obtained images depict the amplitude and frequency variation on time. To obtain the TFR of sEMG signals, CWT was employed, which is a mathematical procedure for analyzing time-varying signals by decomposing a signal into a collection of “wavelets” that can detect time–frequency information [31]. The result, known as a scalogram, is a two-dimensional image of the signal, where the x-axis is the time and the y-axis is the frequency, identifying signal features not visible in time or frequency domains alone. For a signal, s(t), the corresponding CWT is evaluated as:

(1) $${\mathrm{CWT}}_{s(a,b)}=\int s\left(t\right)\varphi_{a,b}^{*}\left(t\right)dt$$

where *a* and *b* are the scale and time value, respectively (a>0, *a* and binR), and $\varphi_{a,b}^{*}\left(t\right)$ is the analyzed mother wavelet defined by the following equation:

(2) $$\varphi_{a,b}^{*}\left(t\right)=\frac{1}{\sqrt{a}}\varphi \left(\frac{t-b}{a}\right)$$

In the present work, CWT was performed using the Morlet wavelet as the mother wavelet, and the scaling value was set to 256. Using CWT to time windows of sEMG signals, a scalogram can be obtained representing the absolute value of the CWT coefficients, with the b and a values along the x- and y-axes, and the magnitude of each point determined from Equation (1). In our approach, the scalograms were retrieved from the time series of the sEMG signals from the Tibialis Anterior and Gastrocnemius Medialis muscles subdivided into 1 s time windows. The algorithmic pipeline was tested with segmentation time windows from 0.5 to 2.5 s in 0.5 s increments. The best performance in distinguishing between normal and toe walking based on sEMG signals was obtained with a time window of 1 s. This choice provides a balance between capturing a sufficient number of muscle activation patterns for reliable classification and maintaining a responsive and computationally efficient system. The corresponding scalograms were downscaled to 224 × 224 to match the input layer of the selected pre-trained architectures described in Section 2.4.

**Figure 4:**
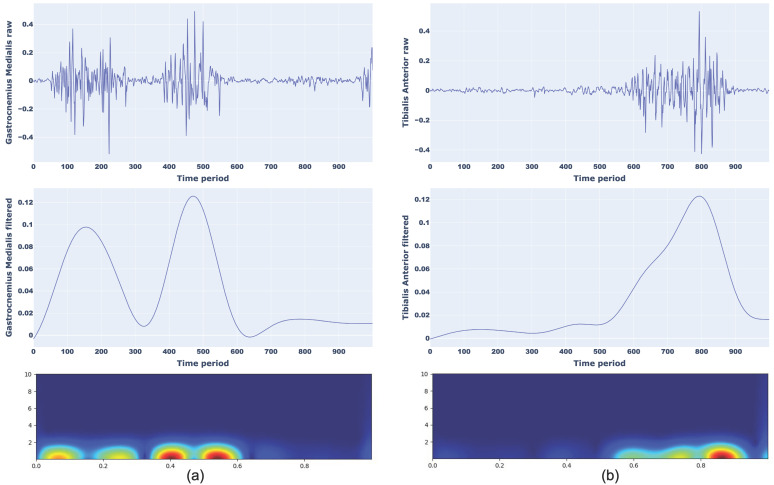
Representation of raw signal, filtered signal, and CWT scalograms for normal walking considering Gastrocnemius Medialis (**a**) and Tibialis Anterior (**b**).

**Figure 5:**
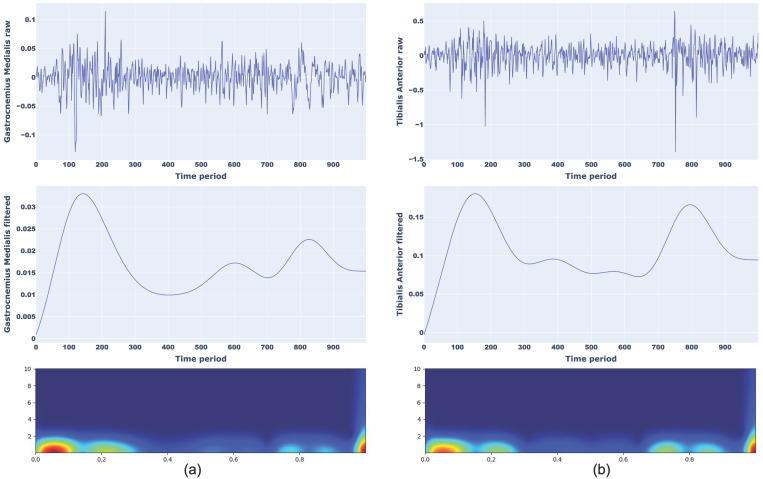
Representation of raw signal, filtered signal, and CWT scalograms for toe walking considering Gastrocnemius Medialis (**a**) and Tibialis Anterior (**b**).

An example of a scalogram of normal walking for Gastrocnemius Medialis (a) and Tibilias Anterior (b) and toe walking for Gastrocnemius Medialis (a) and Tibilias Anterior (b) with corresponding raw and filtered sEMG signals are presented in Figure 4 and Figure 5, respectively.

### 2.4. Classification Approach for Toe Walking

To automatically classify sEMG scalograms in normal and toe walking, TL architectures were used in this work to limit the major problem of CNN architectures, i.e., the large training data requirements. This problem is more marked in the case of complex signals, as with sEMG. To increase learning, the main idea of TL is to transfer gained knowledge [32] from a dataset (i.e., “origin domain”) to a new dataset (i.e., “target domain”). In an EMG signal application context, our major motivation to use TL is the difficulty in identifying the most appropriate features for a correct classification due to the complexity of the signal under investigation.

In this study, six pre-trained models, DenseNet121, MobileNetV3, VGG16, ResNet50, InceptionV3, and InceptionResNetV2, were used to classify toe walking. These models were chosen because they were the most widely used in the analysis of the sEMG signal, although in different contexts from the proposed scenario in this work [33,34,35,36].

DenseNet121 (DN121) [37] is a model specializing in the use of a convolutional neural network in depth, capitalizing on the smallest knowledge between layers, linking with all others located lower in the grid, allowing maximum information flow between all layers. DenseNet121 includes several blocks, such as dense and transition blocks. Specifically, the model contains an input layer followed by a convolution layer, a dense layer, a convolution layer, a pooling layer, another dense layer, a convolution layer, a pooling layer, a dense layer, a pooling layer, and a linear layer, and finally the output.

MobileNetV3 (MN3) [38] is the evolution of MobileNetV1 and MobileNetV2. To improve computational efficiency and extract feature information efficiently, it consists of a deep convolution and a linear bottleneck structure. In this model, 3 × 3 and 5 × 5 convolution kernels are used. Overall, MobileNetV3 networks require less training time due to fewer parameters than large networks.

VGG16 [39] is considered one of the most sophisticated vision models currently available and is simple to use with TL. The number 16 refers to 16 weighted layers. Due to its massive training, this model ensures good accuracy also when the datasets are minimized. VGG16 is a classification model able to classify 1000 unique categories. The network consists of an input layer followed by 2 convolution networks, a max-pooling layer, 3 convolution layers, and another max-pooling layer. Then, the architecture includes 3 convolution layers, 1 max-pooling layer, 3 convolution layers, another max-pooling layer, 3 convolution layers, and another max-pooling layer. Finally, there are 3 dense layers and the output layer.

ResNet50 (RN50) [40] is composed of 48 convolution layers followed by 1 max-pooling layer and 1 average-pooling layer. An important innovation is the use of residual connections, allowing a residual set of functions to map the input to the desired output. The identity mapping method in ResNet enables the model to override a layer if the actual layer is not required. However, this avoids overfitting the training set.

InceptionV3 (Inc3) [41] is an improved version of the InceptionV1 model, factoring the convolutional layers to optimize the number of parameters. Specifically, the model includes three blocks: (1) the basic convolutional block, (2) the enhanced Inception block, and (3) the classifier. The first block, alternating convolutional and max-pooling layers, is responsible for feature extraction. To reduce the number of parameters and the computational overhead, the convolution is decomposed into smaller convolutions. As a result, InceptionV3 has state-of-the-art performance in object recognition and, therefore, this model is largely employed for TL.

InceptionResNetV2 (IncRN2) [42] is a modified version of the InceptionV3 model with improved computing power, increased network depth, and network non-linearity. The feature extraction consists of three similar modules: Inception-ResNet-A, Inception-ResNet-B, and Inception-ResNet-C. In particular, the second and third have asymmetric convolution kernels, as opposed to the symmetric ones of the first.

**Figure 6:**
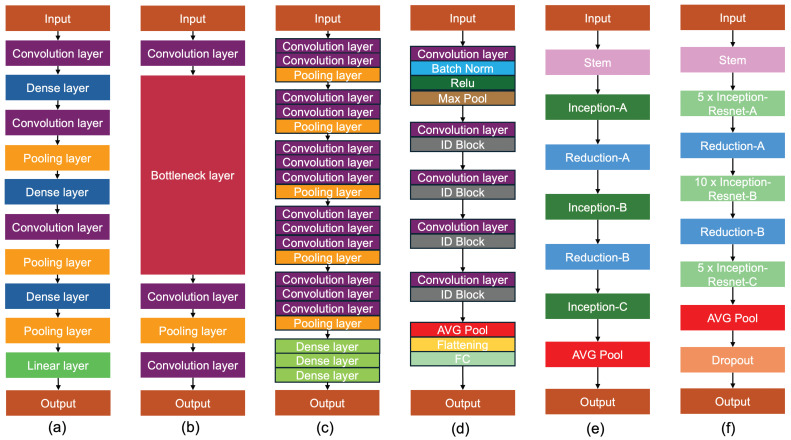
Architecture of six models employed in this study. (**a**) DenseNet121 contains an input layer followed by a convolution layer, a dense layer, a convolution layers, a pooling layer, another dense layer, a convolution layer, a pooling layer, a dense layer, a pooling layer, and a linear layer, and finally the output. (**b**) MobileNetV3 consists of three deep convolution and a linear bottleneck structure. (**c**) VGG16 contains various convolution layers, pooling layers, and dense layers. The number 16 refers to 16 weighted layers. (**d**) ResNet50 is composed of 48 convolution layers followed by 1 max-pooling layer and 1 average-pooling layer. (**e**) InceptionV3 includes three blocks: (1) the basic convolutional block, (2) the enhanced Inception block, and (3) the classifier. (**f**) InceptionResNetV2 consists of three similar modules: Inception-ResNet-A, Inception-ResNet-B, and Inception-ResNet-C, where the second and third have asymmetric convolution kernels, as opposed to the symmetric ones of the first.

All six architectures used in this work are shown in Figure 6.

**Figure 7:**
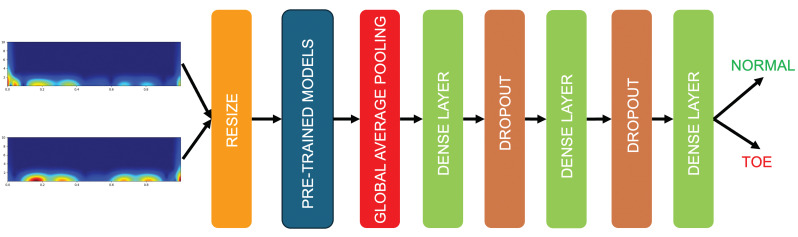
TL architecture implemented in the proposed approach. Resulting scalograms are resized to 224 × 224 to fit with the input layer of the selected deep architectures. Global Average Pooling was appended after the main architectures, followed by various dense layers and dropout layers to obtain the classification of normal and toe walking.

In this work, to classify toe walking, Global Average Pooling was appended after the main architectures to increase the connection of feature importance with the label class [43]. Subsequently, a dense layer, a dropout layer, another dense layer, a dropout layer, and a final dense layer with a “softmax” activation function were added. “Adam” was used as the optimization algorithm, with accuracy as the metric. The implemented architecture is illustrated in Figure 7, while the various network parameters are reported in Table 2.

## 3. Results and Discussion

To evaluate the effectiveness of the proposed approach, several experiments have been performed. The main algorithms were implemented using Python 3.8, with the following libraries: Tensorflow (2.10), pandas (2.0.3), scikit-learn (1.2.1), spkit (0.0.9.6.7). The hardware environment was a Dell^™^ Precision 7920 Rack workstation with 256 GB RAM, dual Intel Xeon Gold 5218R CPU@2.10 Ghz processors, and three NVIDIA^™^ RTX A2000 12 GB GPUs.

Results were analysed using the following four different metrics: accuracy (Acc), precision (Pr), recall (Re), and F1-Score, derived as:

(3) $$\begin{array}{ccc} \mathrm{Acc} & = & \frac{\mathrm{TP}+\mathrm{TN}}{\mathrm{TP}+\mathrm{TN}+\mathrm{FP}+\mathrm{FN}} \end{array}$$

(4) $$\begin{array}{ccc} \mathrm{Pr} & = & \frac{\mathrm{TP}}{\mathrm{TP}+\mathrm{FP}} \end{array}$$

(5) Re=TPTP+FN

(6) $$\begin{array}{ccc} \text{F1-Score} & = & \frac{2*\mathrm{TP}}{2*\mathrm{TN}+\mathrm{FP}+\mathrm{FN}} \end{array}$$

where TP (True Positive) denotes the presence of a toe walking detected successfully by the algorithm; FP (False Positive) implies the absence of toe walking but the algorithm detects it; TN (True Negative) means toe walking is missing and the algorithm correctly does not detect it; and finally, FN (False Negative) denotes toe walking but is not detected by the algorithm. Accuracy displays the ratio of all correctly classified samples to all samples, precision refers to the model’s accuracy in finding positive occurrences, recall refers to the model’s performance in successfully matching positive occurrences using all positive occurrences, and the F1-Score impacts true positive occurrences more than precision. The performance of the proposed approach was evaluated using a 10 cross-validation procedure [44]. Specifically, each pre-trained architecture was trained with 80% of the dataset, while the remaining 20% was used as the test set. Then, to prevent over-fitting, a validation set was generated with 20% of the training set. To reduce the simultaneous occurrence of the same samples in the training and test sets, the procedure was performed 10 times, with different training and test sets.

Table 3 reports the achieved results for each considered TL architecture. The metrics considered and the training time on the previously described hardware are included. From Table 3, it can be seen that the modified InceptionResNetV2 architecture performed best in terms of average accuracy compared to the others. In fact, after training 150 epochs, it achieved an accuracy of 95.25%, which is higher compared to the other evaluated architectures. In terms of computational efficiency, a comparable training time can be seen for all the proposed architectures, with the exception of modified MobileNetV3, which has a lower training time of about 30–40%, but a much worse accuracy. Considering the two best performing architectures (InceptionResnetV2 and DenseNet121), the difference in training time is about 280 s, which does not represent a significant challenge for actual hardware capabilities.

For the model with the best performance (InceptionResNetV2), Table 4 also shows the obtained performance for various considered users. For the sake of brevity, we report the considered metrics for six users with the anthropometric characteristics reported in Table 5. As we can see from the achieved results, the model continues to perform well, maintaining a similar accuracy for all the users whose Gender, Age, Body Height, and Body Mass are quite different from each other, confirming the goodness of the proposed approach.

**Figure 8:**
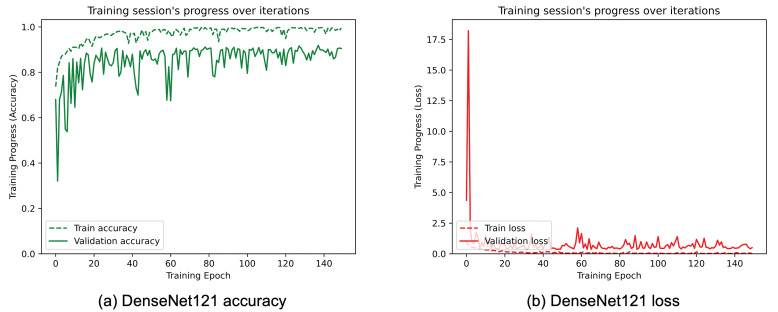
(**a**) Accuracy and (**b**) loss of the proposed DenseNet121 at the training and validation phase.

**Figure 9:**
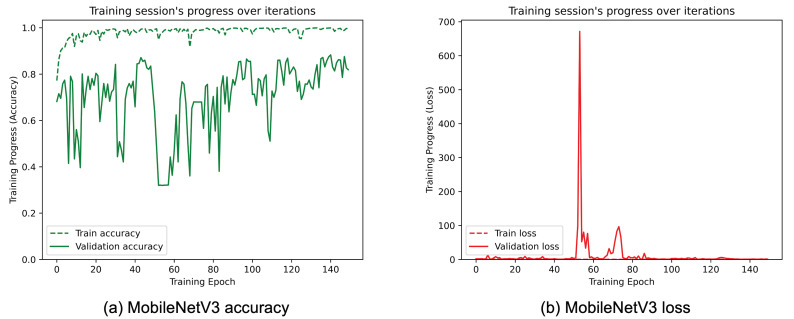
(**a**) Accuracy and (**b**) loss of the proposed MobileNetV3 at the training and validation phase.

**Figure 10:**
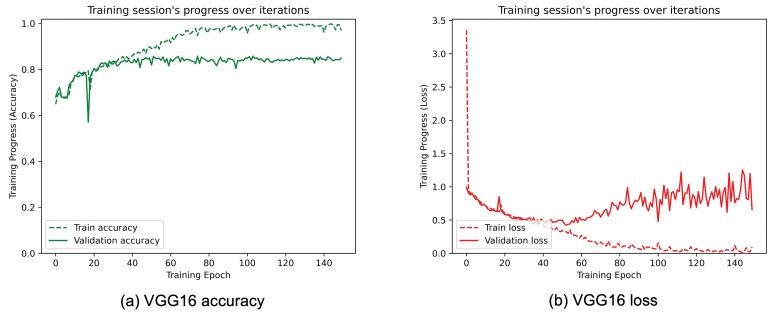
(**a**) Accuracy and (**b**) loss of the proposed VGG16 at the training and validation phase.

**Figure 11:**
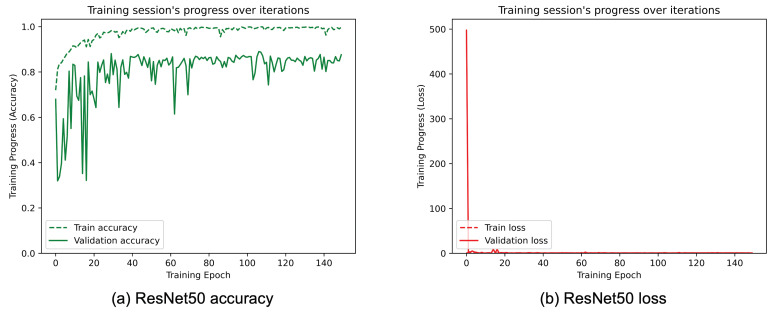
(**a**) Accuracy and (**b**) loss of the proposed ResNet50 at the training and validation phase.

**Figure 12:**
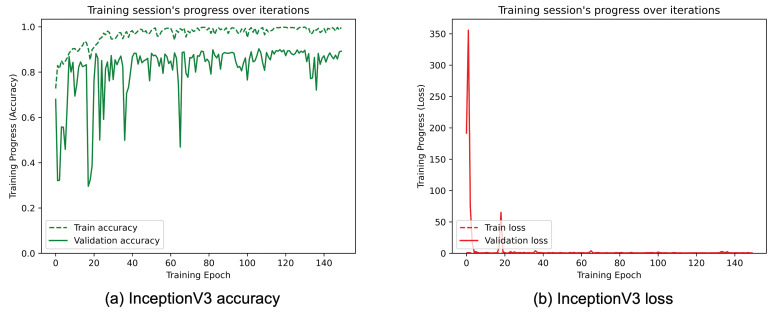
(**a**) Accuracy and (**b**) loss of the proposed InceptionV3 at the training and validation phase.

**Figure 13:**
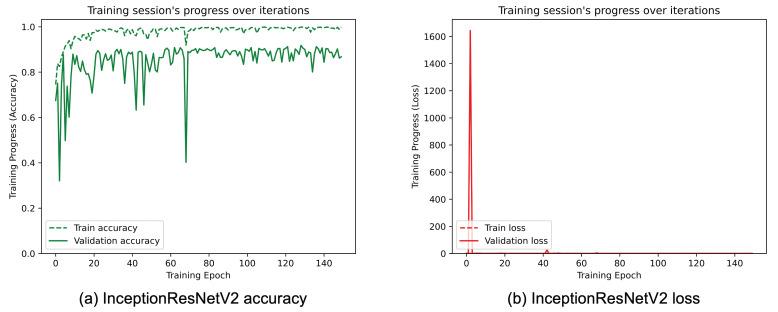
(**a**) Accuracy and (**b**) loss of the proposed InceptionResNetV2 at the training and validation phase.

Figure 8, Figure 9, Figure 10, Figure 11, Figure 12 and Figure 13 show the overall models’ performance, displaying model losses and accuracy in training and validation. From these figures, it can be seen that the validation accuracy can hardly be improved and oscillates around a robust value after 150 epochs of training, although the training accuracy constantly improves. Differences between training and validation accuracy and loss can also be noted, probably related to over-fitting problems most visible in the MobileNetV3, VGG16, and ResNet50 models, i.e., the models with the worst performance (90% lower accuracy). However, again from these figures it can be seen that the best model is still the modified InceptionResNetV2, given the smaller difference in accuracy between the training and validation phases, while MobileNetV3 is still the worst model, as can be seen in Figure 9a, given the large difference in the two phases.

**Figure 14:**
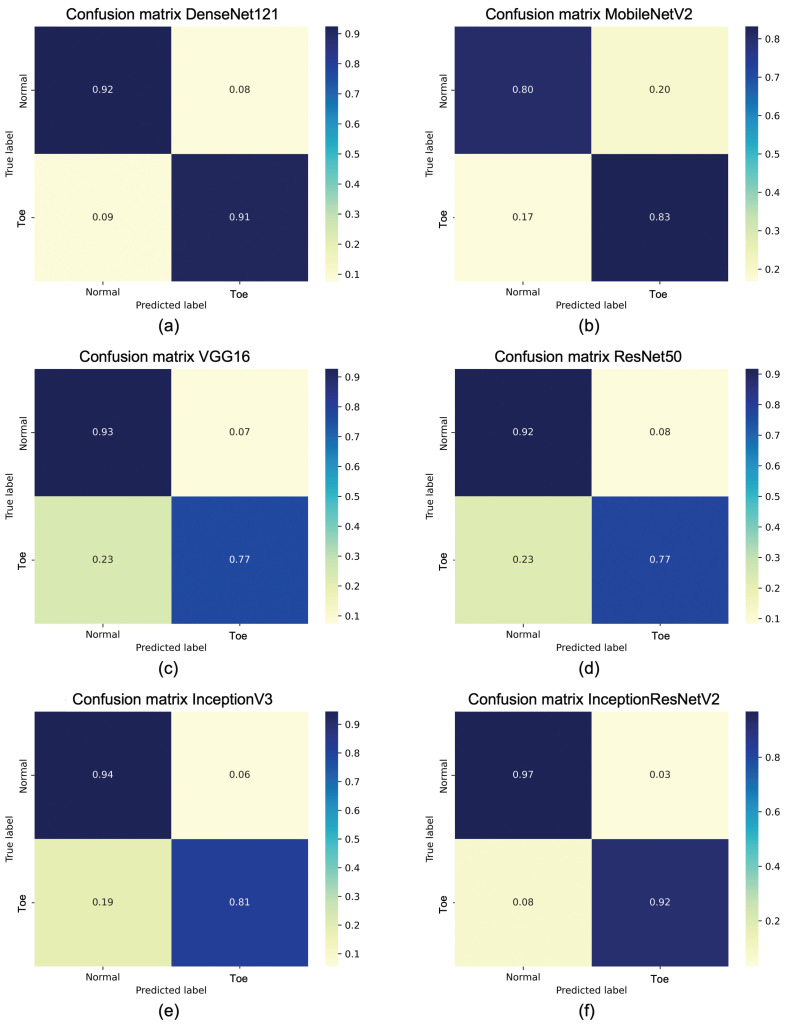
The confusion matrix on test dataset for (**a**) DenseNet121, (**b**) MobileNetV2, (**c**) VGG16, (**d**) ResNet50, (**e**) InceptionV3, (**f**) InceptionResNetV2.

Figure 14 shows the confusion matrices of the obtained average accuracies for each considered model. Again, observing the numbers on the main diagonal, modified InceptionResNetV2 remains the best performing model, followed by DenseNet121, while, once again, MobileNetV2 performs the worst. In addition, the confusion matrices reported for each considered architecture show that, for the best models, normal and toe walking both obtain an accuracy greater than 90% and similar to each other. In particular, InceptionResNetV2 achieves an accuracy of 97% for normal walking. Finally, it is evident that the lowest accuracy is found in the toe walking classification for VGG16 and ResNet50, which is 77%.

Once the best model was identified, it was saved in “.h5” format and, to show the real generalization of the proposed approach, it was tested using the FREEEMG1000 system, as described in Section 2.1. Table 6 reports the obtained results for the considered metrics varying the five considered users. It can be seen that, again, the proposed approach appears to be promising, although with a small dataset. In fact, in this case, an average accuracy of about 93.4% is obtained.

Finally, three classical Machine-Learning (ML) classifiers were used to further validate the proposed approach, using both the public dataset and the dataset obtained using the BTS system. In particular, Support Vector Machine (SVM) [45], Random Forest (RF) [46], and K-Nearest Neighbor (KNN) [47] were considered. The obtained results are shown in Table 7 and Table 8, respectively. In particular, considering the best model with the proposed approach (InceptionResNetV2) and the one with ML (RF), an increase of about 6% can be seen with the proposed approach using the public dataset and about 8% with the BTS system, which represents a considerable improvement in performance, even though it requires a significantly higher training time. However, the training is only performed once offline, and therefore the higher training time does not affect the total processing time during continuous monitoring. This ensures real-time operation in normal and toe walking classification using the previously trained model, also on embedded platforms such as Odroid N2+ [48]. This platform has a limited cost (around EUR 200) and is also equipped with a GPU, which certainly makes it usable in real environment contexts. In support of this, some tests have been carried out on Odroid N2+, achieving an average time to obtain the classification in normal or toe walking of approximately 0.114 s by giving, as input to the model, the scalograms acquired by the two muscles in the analyzed window.

## 4. Conclusions

Persistent toe walking can lead to foot and ankle muscle issues, resulting in instability, pain, and an increased risk of falls. Moreover, such walking can adversely influence quality of life, making it difficult, for example, to participate in sports activities or, even worse, provoking teasing and bullying in children. So, early detection can prevent more serious health problems, such as Achilles tendon shortening, avoiding negative impacts and consequently improving quality of life. Consequently, an accurate, safe, and low-cost detection method for toe walking is very important.

In this study, a novel approach for detecting toe walking using surface electromyography on the lower limb is proposed. sEMG sensors can facilitate early detection of the problem by measuring muscle electrical activity, enabling the identification of signals prior to the corresponding movement associated with muscle activation. To reduce the complexity of the sEMG signal and enhance generalization across different devices, a TL approach is introduced. Scalograms were applied to track the data in the time–frequency domain, attenuating the sEMG signal constraints. Six different pre-trained architectures were used to distinguish toe walking from normal walking, and the best results were obtained with InceptionResNetV2, achieving an average accuracy of about 95.2% on a public dataset. The approach was also validated using a different sEMG device from the one employed in the public dataset. Specifically, participants were asked to perform normal walking and toe walking tasks, generating a small dataset with the new device. The positive results obtained demonstrate the system’s ability to generalize across different devices.

Current sEMG systems, despite their effectiveness, exhibit certain limitations, such as the challenge of precisely positioning probes on targeted muscles and the discomfort that can arise from prolonged use. To address these concerns, an innovative approach for future development could involve the creation of sensorized wearable garments—such as socks, sleeves, or bands—that seamlessly integrate the necessary electronic and software components. These advanced garments would facilitate accurate probe placement while employing hypoallergenic materials designed to minimize skin irritation, thereby enhancing overall user experience. This approach would also reduce the requirement for specialized personnel, significantly improving user convenience and comfort during extended monitoring periods. Moreover, future advancements will likely focus on building an extensive dataset through collaboration with other project partners, enriching the quality and applicability of the research. Given the overfitting observed in some of the analyzed models, such as MobileNetV3, VGG16, and ResNet50, employing strategies like Regularization or Data Augmentation—potentially using Generative Adversarial Networks—will be essential to mitigate this issue. Further exploration of additional pre-trained architectures will be pursued to determine if InceptionResNetV2 remains superior. Additionally, expanding the scope of analysis to include other walking disorders, such as heel walking, and investigating additional muscles will provide a more comprehensive understanding of sEMG applications.

## Figures and Tables

**Table 1:** Summary of related studies on toe walking.

References	Sensor Type	Methodology	Accuracy
[12]	accelorometer	k-means	0.96
[13]	accelerometer	Machine Learning	0.93
[14]	accelerometer and gyroscope	Machine Learning	0.93
[15]	accelerometer and gyroscope	Deep Learning	0.93
[16]	accelerometer	Machine Learning	0.87
[17]	accelerometer and gyroscope	Deep Learning	0.94

**Table 2:** Hyperparameters for the proposed TL models.

Hyperparameter	Model Architecture					
	DN121	MN3	VGG16	RN50	Inc3	IncRN2
Learning rate	0.001	0.001	0.002	0.001	0.002	0.002
Batch size	128	128	128	128	128	128
Optimizer	Adam	Adam	Adam	Adam	Adam	Adam
Output activation	softmax	softmax	softmax	softmax	softmax	softmax
# epochs	150	150	150	150	150	150

**Table 3:** Comparison of the performance for each TL architecture.

Model	Accuracy	Precision	Recall	F1-Score	Training Time (s)
DenseNet121	0.9200	0.9221	0.9179	0.9206	5571.04
MobileNetV3	0.8372	0.8720	0.8636	0.8417	3595
VGG16	0.8739	0.8725	0.8479	0.8725	6095.78
ResNet50	0.8692	0.8678	0.8447	0.8681	4869.20
InceptionV3	0.9007	0.9004	0.8791	0.9002	4845.21
InceptionResnetV2	0.9525	0.9519	0.9440	0.9519	5849.34

**Table 4:** Performance comparison of InceptionResNetV2 for different users.

Users	Accuracy	Precision	Recall	F1-Score
Subject28	0.9595	0.9523	0.9468	0.9506
Subject30	0.9518	0.9571	0.9463	0.9572
Subject22	0.9438	0.9486	0.9452	0.9479
Subject20	0.9586	0.9512	0.9437	0.9511
Subject10	0.9532	0.9494	0.9428	0.9507
Subject21	0.9507	0.9512	0.9443	0.9514

**Table 5:** Anthropometric characteristics of the considered users.

Subject	Gender	Age (Years)	Body Height (cm)	Body Mass (kg)
Subject28	M	7	118.786141	29.037024
Subject30	F	9	134.932471	32.973236
Subject22	M	32	174.447799	70.764818
Subject20	F	34	155	51.625319
Subject10	M	56	179.569248	74.179373
Subject21	F	68	154.322319	55.007142

**Table 6:** Performance for each users using BTS FREEEMG1000.

End-User	Accuracy	Precision	Recall	F1-Score
User 1	0.9518	0.9512	0.9447	0.9512
User 2	0.9198	0.9201	0.9196	0.9198
User 3	0.9437	0.9451	0.9438	0.9432
User 4	0.9070	0.9103	0.9079	0.9071
User 5	0.9460	0.9540	0.9370	0.9410

**Table 7:** Obtained performance with ML classifiers using public dataset.

Model	Accuracy	Precision	Recall	F1-Score
SVM	0.8412	0.8418	0.8412	0.8414
RF	0.8904	0.8904	0.8904	0.8904
KNN	0.8701	0.8700	0.8701	0.8698

**Table 8:** Obtained performance with ML classifiers using dataset with BTS.

Model	Accuracy	Precision	Recall	F1-Score
SVM	0.8116	0.8122	0.8115	0.8109
RF	0.8644	0.8604	0.8604	0.8604
KNN	0.8576	0.8579	0.8567	0.8577

## Data Availability

The data presented in this study are available on request from the corresponding author. The data are not publicly available due to restrictions (with them containing information that could compromise the privacy of research participants).
